# CT-Guided Adaptive Radiotherapy for the Treatment of Technically Challenging Oligometastatic Disease: A Case Report

**DOI:** 10.7759/cureus.70765

**Published:** 2024-10-03

**Authors:** Peter L Lee, Xiaoming Chen, Ahmed ElDib, Thomas J Galloway, Mark A Hallman, CM Charlie Ma, Joshua E Meyer, Rebecca M Shulman

**Affiliations:** 1 Department of Radiation Oncology, Fox Chase Cancer Center, Philadelphia, USA

**Keywords:** adaptive radiation therapy, cone-beam computed tomography (cbct), ethos, intrafraction motion, oligometastatic tumors, sbrt (stereotactic body radiotherapy), stereotactic ablative radiation

## Abstract

There is a growing interest in the application of stereotactic body radiotherapy (SBRT) for the treatment of oligometastatic cancers. This increasing appeal of SBRT has highlighted the need for more sophisticated radiotherapy techniques that allow high doses of radiation to be delivered to multiple sites while limiting the exposure of neighboring healthy tissue. A major obstacle to achieving this aim has been the occurrence of interfraction target variability: the tendency of both the tumor and the surrounding tissue to undergo day-to-day non-synchronous shifts in position. Such changes in the conformation of the tumor field often compromise the effectiveness of conventional SBRT prescribed for a fixed target. We report a case of oligometastatic pelvic disease where the challenge of an unusually mobile tumor was overcome with the use of a novel technique employing cone beam CT (CBCT)-based online adaptive radiotherapy (OART).

The Phase I “Adaptive Radiation for Abdominopelvic Metastases (ARAM)” clinical trial was designed to determine if OART can achieve dosing targets superior to those attained using conventional radiotherapy techniques. In this case, CT adaptive planning enabled the treatment of a pelvic target prescribed per protocol to 45Gy that would otherwise have not been amenable to treatment with conventional SBRT planning. Adaptive plans showed significant improvements in target coverage while respecting critical organ constraints, resulting in a total treatment V35Gy of 89.3% and V45Gy of 52.8%, whereas the scheduled plan would have achieved V35Gy of 67.4% and V45Gy of 13.6%. Treatment times were variable (38.1-96.7 mins), and correlated with the magnitude of daily translation which ranged from 4 to 7 cm of total linear translation. The patient tolerated treatment without any adverse events. These results demonstrate a novel application of CBCT-guided OART that allowed for the administration of ablative treatment to an unexpectedly mobile target unamenable to conventional SBRT. CBCT-guided OART currently requires increased treatment time, a need which might be reduced by optimization of daily contouring. The phase 1 clinical trial NCT05880667 is ongoing and may provide further evidence that CBCT-guided OART can meet the technical challenges posed by radiotherapy for oligometastatic abdominal and pelvic disease.

## Introduction

Several randomized trials have reported that stereotactic body radiotherapy (SBRT) can be employed in the setting of oligometastatic disease to achieve improved survival and longer delays before the initiation of systemic therapy [1-5]. Additional trials further exploring the potential role of SBRT in this setting are ongoing [6-9]. A critical requirement, however, is the ability to deliver fractional doses of radiation to a conformal volume with biologic equivalent dose (BED) goals of >75Gy [10]. In fact, other recent data suggest goals of >100Gy or even >125Gy to be increasingly effective [11,12]. This requirement has inspired several technical solutions, including traditional coplanar gantry-based linear accelerators (LINACs), non-coplanar gantry systems, and robotic arm LINACs. While all of these techniques have merit, they do not address the challenge posed by cases with significant day-to-day positional variability of the target and surrounding critical organs.

Daily image-guided online adaptive radiotherapy (OART) has emerged as a promising technique that allows superior coverage of target volumes when significant intrafractional motion is anticipated [13,14]. OART may be delivered using either MR-based or cone beam CT (CBCT)-based daily imaging. The adaptive workflow allows for daily review and, if necessary, real-time re-planning so that ablative radiation treatment can be delivered within dose constraints. Studies examining the clinical utility of both MR- and CT-based modalities are ongoing [15-17].

In this report, we describe a challenging case involving the treatment of oligometastatic disease in the pelvis. The target was shown to undergo significant positional variability between CT and MRI simulation scans, ruling out the use of ablative therapy with traditional SBRT. The availability of CBCT-guided OART, however, allowed us to treat this variable target to an ablative dose, while adequately sparing surrounding structures. We additionally report dosimetric comparisons between adaptive and scheduled plans and address the logistical considerations of adaptive planning.

## Case presentation

An 84-year-old male presented for consultation regarding radiotherapy to treat a metastatic gastrointestinal stromal tumor (GIST). He had been initially diagnosed in 2016 with GIST of the cecum requiring surgical resection followed by an adjuvant tyrosine kinase inhibitor, which had been discontinued after three years due to adverse effects. In 2021, he had been found to have new liver metastasis and restarted on a tyrosine kinase inhibitor. He had then again remained stable until his recent presentation with a right lower quadrant peritoneal metastasis, which had increased in size over two months from 2.2 x 2.8 cm to 3.8 x 2.9 cm.

At the time of his evaluation, the patient ambulated independently with an Eastern Cooperative Oncology Group (ECOG) performance status of 1. After a discussion with the patient, he was enrolled in clinical trial NCT05880667 and scheduled for fiducial marker placement and treatment of his enlarging pelvic metastasis.

Clinical trial

NCT05880667 is a single-institution phase I trial employing adaptive stereotactic body radiation to treat abdominopelvic metastases. Its primary objective is to assess the ability of CT-based adaptive SBRT to deliver increased doses of radiation safely to metastatic disease posing significant technical challenges. Bayesian Optimal Interval (BOIN) design will be used to define dose escalation and de-escalation rules based on the proportion of patients experiencing dose-limiting toxicity (DLT). Eligibility criteria include confirmed cancer that is metastatic with at least one target lesion in the abdomen or pelvis. All targeted lesions must be ≥10 mm. Patients must have an ECOG performance status of 0 or 1 and an estimated survival of ≥12 months. Patients will be followed up for up to five years, with endpoints including rates of local control, overall survival (OS), progression-free survival (PFS), and patient-reported outcomes. Acute and late toxicities will be recorded.

Treatment planning and delivery

**Figure 1:**
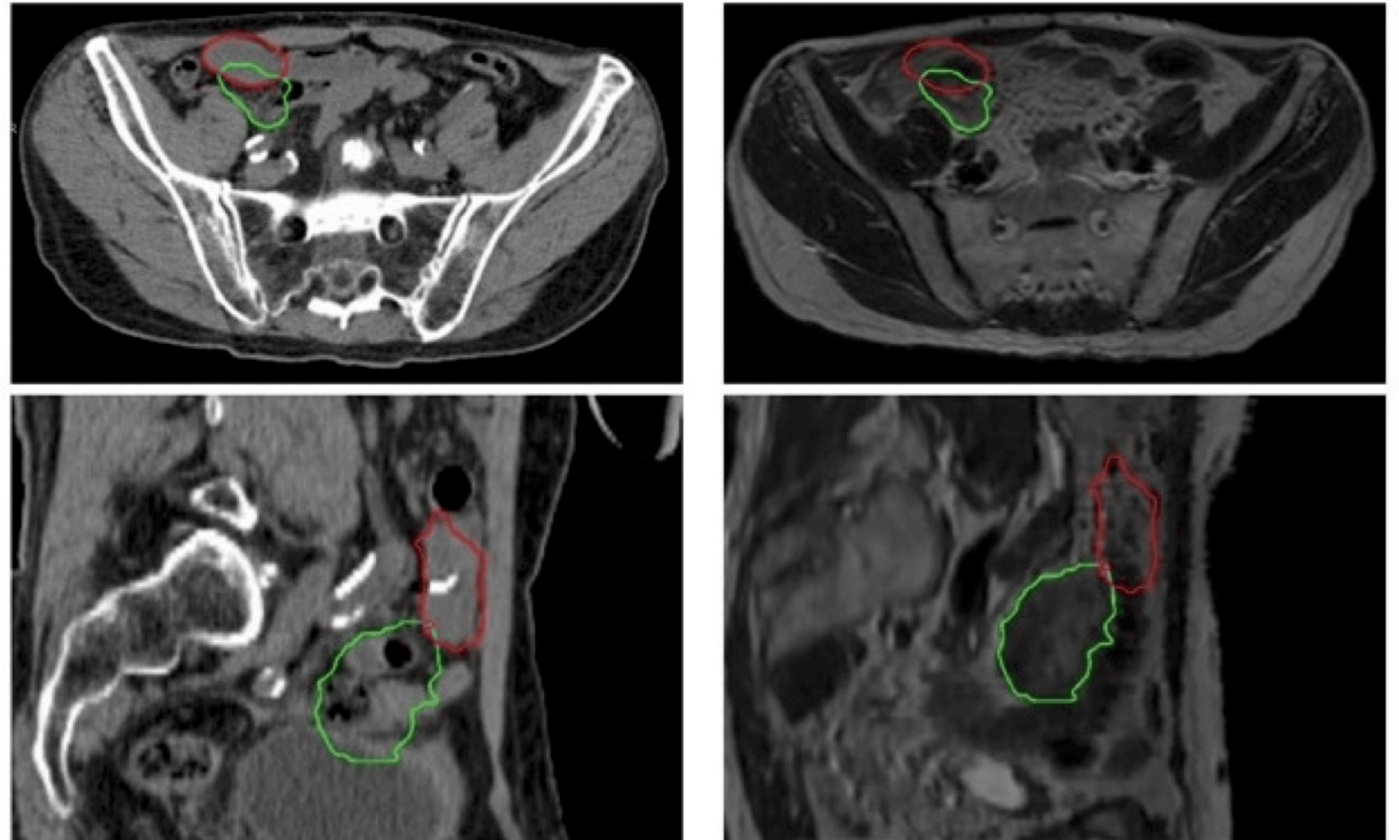
CT and MRI simulation Representative axial and sagittal slices from CT (left) and MRI (right) simulation scans. The red outline represents GTV determined from CT. The green outline represents GTV determined from the MRI CT: computed tomography; GTV: gross tumor volume; MRI: magnetic resonance imaging

The patient had two gold fiducial markers placed in the right lower quadrant peritoneal mass about one week before his scheduled simulation. At the time of fiducial marker placement, his mass increased in size from 3.8 cm to 4.8 cm. For simulation, a custom immobilization device was created for the patient while lying supine, with arms above the head. CT simulation and MRI simulation scans were obtained with the patient in the treatment position. Fluoroscopic imaging was obtained as well as a free-breathing 4D-CT scan to assess for tumor motion during the breath cycle. Motion of the fiducials was measured and found to be negligible throughout the breath cycle. It was determined that motion management techniques such as abdominal compression, respiratory gating, or internal target volume (ITV) generation were not necessary for this target. Significant intrafraction motion relative to the breath cycle was not anticipated for this particular target. Gross tumor volume (GTV) was determined using simulation and diagnostic imaging. As shown below in Figure 1, there was a significant change in the location of the target between CT simulation and MRI simulation acquisitions. In sum, the simulation scans indicated a high likelihood of interfraction motion and minimal intrafraction motion as related to the breath cycle.

Given the linear and rotational changes noted between CT and MRI simulation scans, the location of GTV was determined with the CT, while MRI was used to aid in the delineation between the tumor and the surrounding bowel. Registration of the CT and MRI simulation scans was not done due to the large positional shift. Rather, the CT simulation was used as the primary contouring sequence, while the MRI was utilized to help delineate borders between the target and organs at risk (OARs). Of note, the tumor was relatively dense compared to surrounding tissue, which allowed for adequate visualization on CT and CBCT imaging. A symmetric 3 mm planning target volume (PTV) expansion was used upon the GTV per clinical trial protocol. No ITV was generated as the motion of the fiducials on free breathing 4D-CT simulation was noted to be negligible.

Dose constraints to critical structures are listed in Table 1 below; these were used both for the initial plan and subsequent adaptive plans. Per protocol, PTV was prescribed to 45Gy, although coverage was sometimes compromised in order to meet OAR constraints. An additional structure, PTV_cropped, was generated where any PTV volume extending into an OAR was cropped; no margin or gradient was applied. A clinical planning objective was applied to the PTV_cropped volume shown in the table below. On each treatment day, initial GTV and OAR contours were automatically registered by the treatment planning software to the current CBCT (acquired using HyperSight CBCT). These structures are standardly deformably propagated. These were reviewed, edited, and re-contoured if necessary at the discretion of the day’s reviewing physician and physicist. No contour ring was utilized. An adaptive plan was then generated and optimized. Intensity-modulated radiation therapy (IMRT) plans were generated with nine equidistant fields, which are pre-defined by the planning software per protocol. At the same time, the scheduled plan was re-calculated based on the day’s anatomy. These two plans were evaluated and compared, and a decision was made by the covering physician to use either the scheduled or adapted plan per clinical goals and priority. Plan adaptation was done with normal tissue dose as the driver of plan acceptability. All target coverage goals may not have been met to meet normal tissue dose constraints.

**Table 1:** Clinical goals and dose constraints in the order of priority OARs: organs at risk; PTV: planning target volume

Clinical goals and the order of priority	
(Top = highest priority; bottom = lowest priority)	
OARs proximal to the target. Clinical goals required to be met during adaptation	
Skin	D0.03cc <38.5Gy
Small bowel	D0.03cc <40Gy
Large bowel	D0.03cc <40Gy
Target volume. Clinical goals may not be met during adaptation	
PTV (exclude the overlap with OARs)	V45Gy ≥96%
		PTV	V45Gy ≥95%
OARs distal to the target. Clinical goals will be met during adaptation	
Bladder	D0.03cc <42Gy
Cauda equina	D0.03cc <25Gy
Rectum	D0.03cc <55Gy
Rectum	V50Gy <3.5cc

Results

**Figure 2:**
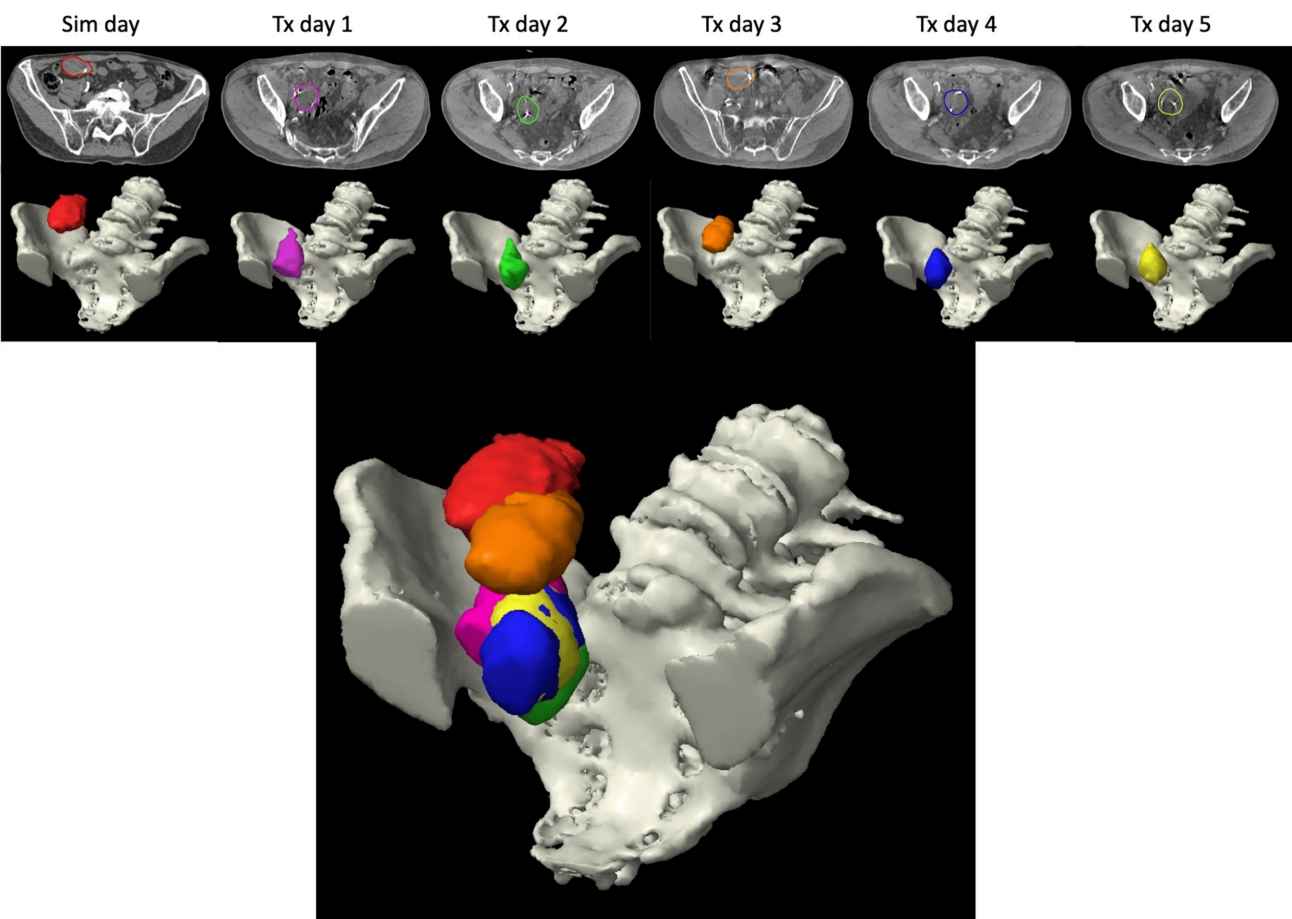
Daily GTVs Daily GTVs re-contoured based on CBCT, compared to initial GTV based on CT sim. Red: CT sim. Pink: treatment day one. Green: treatment day two. Orange: treatment day three. Blue: treatment day four. Yellow: treatment day five CBCT: cone beam computed tomography; GTV: gross tumor volume

The patient received five fractions as scheduled, without acute treatment limiting toxicity. Daily CBCT review demonstrated significant daily linear and rotational movement of the target and OARs. Axial slices of the GTV and fiducial markers can be seen below in Figure 2, as well as daily and composite 3D reconstruction of the GTV.

**Figure 3:**
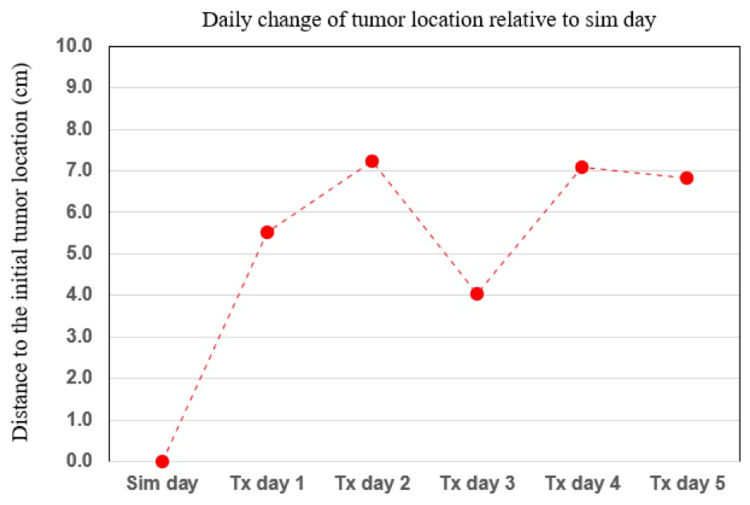
Daily change of tumor location Root sum square of X, Y, and Z linear translations of GTV center of mass, relative to CT simulation CT: computed tomography; GTV: gross tumor volume

The total daily shift in location of the center of mass of GTV was calculated by taking the root sum square of linear translations in the X, Y, and Z directions. This is shown in Figure 3 below.

The mean time on the table per fraction was 57.8 min, with a standard deviation (SD) of 24.2 min. The majority of treatment time was attributed to the initial CBCT review and contouring (mean: 30.4 min), followed by the second CBCT (verifying CBCT) review and treatment delivery (mean: 18.0 min). Of note, alignment to the second CBCT did require shifts for re-alignment, typically ranging between 5 and 10 mm of linear translation. We did not have a rotational couch to allow pitch, yaw, or tilt. 

Time spent on initial CBCT review and re-contouring had the greatest variability, with SD of 21.7 min, while plan generation, QA, and treatment delivery were generally stable with SDs of 0.1 min, 1.9 min, and 3.7 min, respectively. Fractions two and five were treated by a covering physician, while fractions one, three, and four were treated by the initial prescribing physician. Of note, the time spent for contouring of the second fraction was substantially longer than the other fractions (67 min). Overall treatment times and components for each fraction are shown in Table 2.

**Table 2:** Adaptive planning steps and time spent Minutes required for each step of adaptive planning workflow by fraction, as well as mean and SD CBCT: cone beam computed tomography; SD: standard deviation

Fraction #	1st CBCT and contouring time (min)	Plan calculation (min)	Plan review (min)	Physics QA (min)	2nd CBCT review and Delivery time (min)	Total time (min)
							1	18.0	1.8	0.9	5.9	18.4	45.0
2	67.0	1.9	3.7	3.0	21.1	96.7
3	15.8	1.8	0.9	5.8	13.8	38.1
4	17.4	2.0	1.4	8.1	14.5	43.4
5	33.9	2.0	3.7	4.4	22.0	66.0
Mean	30.4	1.9	2.1	5.4	18.0	57.8
SD	21.7	0.1	1.5	1.9	3.7	24.2

Target coverage was significantly greater on the adaptive plan compared to the scheduled for all fractions, with adaptive V35Gy ranging from 77.2 to 94.7% compared to scheduled V35Gy ranging from 60.8 to 82.8%. PTV coverage to the per protocol 45Gy prescription dose was difficult to achieve even with adaptive planning; however, adequate coverage to the more traditional oligometastatic prescription doses of 30-35Gy was manageable, achieving adaptive V35Gy of 89.3% for the complete treatment. Tables 3-4 below show absolute dose coverage to the PTV and PTV_cropped volumes for each fraction per adaptive and scheduled plans.

**Table 3:** PTV coverage Comparison of PTV covered to respective doses between adapted and scheduled plans PTV: planning target volume; SD: standard deviation

Adapted plan					Scheduled plan				
PTV					PTV				
Fx #	V30Gy	V35Gy	V40Gy	V45Gy	Fx #	V30Gy	V35Gy	V40Gy	V45Gy
1	99.9%	93.3%	76.2%	60.5%	1	77.2%	62.5%	41.0%	9.0%
2	99.9%	87.7%	60.5%	45.1%	2	73.3%	60.8%	29.8%	1.7%
3	99.8%	94.7%	80.4%	65.2%	3	95.2%	82.8%	68.5%	47.5%
4	99.9%	93.6%	76.1%	59.9%	4	78.8%	64.1%	36.0%	4.4%
5	97.9%	77.2%	47.2%	33.4%	5	83.0%	67.0%	37.8%	5.2%
Mean	99.5%	89.3%	68.1%	52.8%	Mean	81.5%	67.4%	42.6%	13.6%
SD	0.9%	7.3%	13.9%	13.2%	SD	8.4%	8.9%	15.0%	19.2%
Max	99.9%	94.7%	80.4%	65.2%	Max	95.2%	82.8%	68.5%	47.5%
Min	97.9%	77.2%	47.2%	33.4%	Min	73.3%	60.8%	29.8%	1.7%

**Table 4:** PTV_cropped coverage Comparison of PTV_cropped (PTV excluding overlap with OARs) covered to respective doses between adapted and scheduled plans OARs: organs at risk; PTV: planning target volume; SD: standard deviation

Adapted Plan					Scheduled Plan				
PTV_cropped (excluding overlap with OARs)					PTV_cropped (excluding overlap with OARs)				
Fx #	V30Gy	V35Gy	V40Gy	V45Gy	Fx #	V30Gy	V35Gy	V40Gy	V45Gy
1	100.0%	99.3%	88.5%	70.6%	1	79.7%	65.7%	43.8%	10.1%
2	100.0%	99.2%	81.4%	60.9%	2	76.9%	66.2%	33.3%	1.9%
3	100.0%	99.3%	90.5%	73.5%	3	95.3%	85.8%	73.2%	52.2%
4	100.0%	99.2%	87.4%	68.8%	4	82.8%	68.9%	40.0%	4.8%
5	99.8%	98.6%	74.8%	52.9%	5	94.4%	82.8%	50.0%	6.9%
Mean	100.0%	99.1%	84.5%	65.3%	Mean	85.8%	73.9%	48.1%	15.2%
SD	0.1%	0.3%	6.4%	8.4%	SD	8.5%	9.6%	15.3%	20.9%
max	100.0%	99.3%	90.5%	73.5%	max	95.3%	85.8%	73.2%	52.2%
min	99.8%	98.6%	74.8%	52.9%	min	76.9%	65.7%	33.3%	1.9%

**Figure 4:**
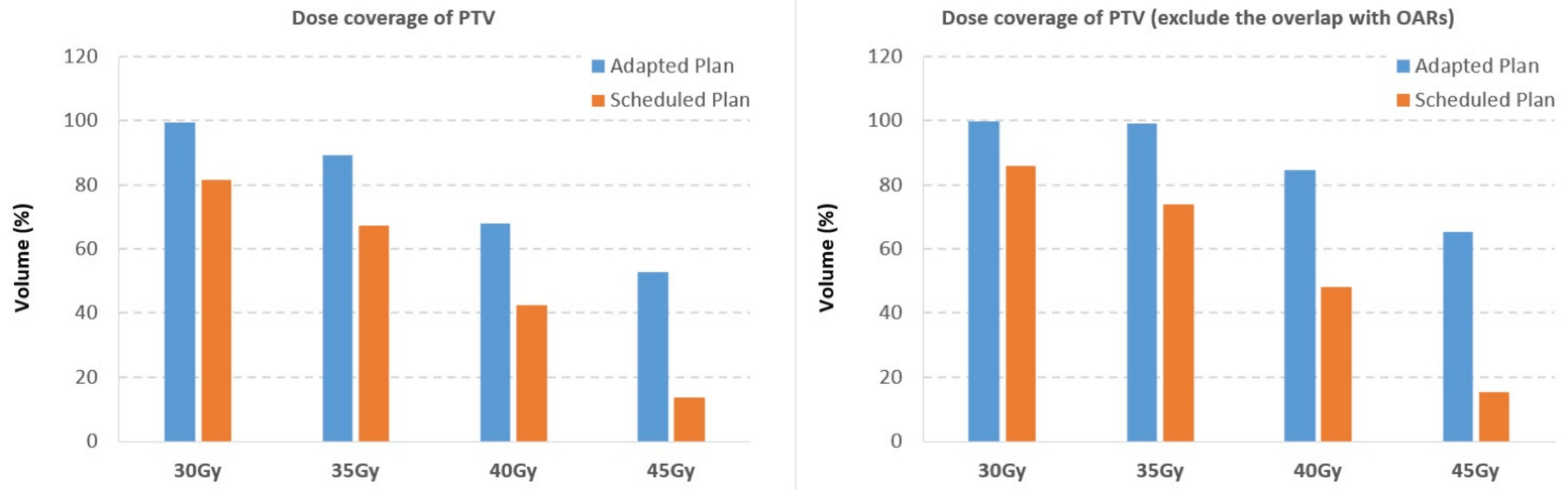
PTV treatment coverage Total treatment PTV and cropped PTV coverage for respective doses, comparing the use of five adaptive fractions versus five scheduled fractions PTV: planning target volume

Ultimately, target coverage by adaptive planning achieved V35Gy and V45Gy of 89.3% and 52.8%, respectively, for the total treatment course. Comparative coverage with scheduled plans for V35Gy and V45Gy were 67.4% and 13.6%. Figure 4 below shows total treatment coverage of PTV.

**Figure 5:**
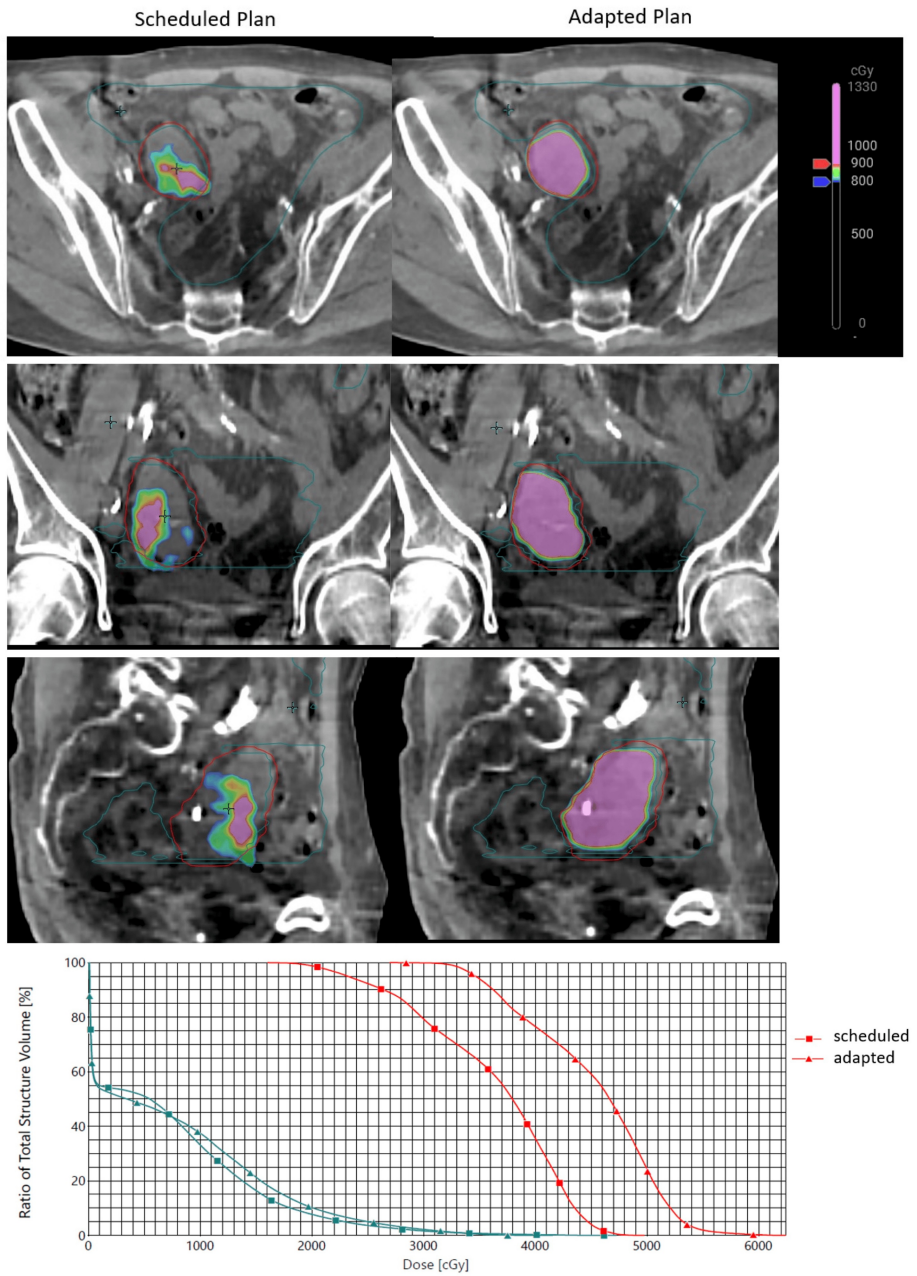
Comparison of scheduled and adaptive plans on fraction four Dose distribution on representative axial, coronal, and sagittal slices, obtained during the fourth fraction of treatment. The red contour represents the daily PTV; the blue contour represents the daily bowel bag. DVH (dose re-scaled to five fractions) of PTV (red) and bowel bag (blue), with square points corresponding to scheduled plan, and triangles to the adaptive plan DVH: dose-volume histogram; PTV: planning target volume

An example of a scheduled plan compared to an adaptive plan dose distribution from fraction four is shown below in Figure 5, as well as the accompanying dose-volume histogram (DVH) of PTV and bowel bag.

## Discussion

We describe a case of oligometastatic disease where the use of CBCT-guided OART allowed for ablative radiation treatment to a target that demonstrated significant interfraction motion. This large shift in position was initially unexpected given the intrapelvic location of the target, as well as a lack of any significant breath cycle-related motion as measured on a free-breathing 4D CT simulation scan. The magnitude of the observed shift of the GTV between fractions would normally have excluded SBRT as a treatment option. Daily CBCT imaging subsequently disclosed that the center of mass of the GTV deviated as much as 7 cm from the CT simulation, and never less than 4 cm (Figure 4). Intrafraction motion was present on treatment days as demonstrated by shifts required for alignment of the second CBCT falling in the 5-10 mm range. This motion was likely attributed to the movement of the patient on the table, as generally larger shifts occurred on days that required longer re-planning times, specifically day two. In total, however, the motion of the target in this case was largely realized on an interfraction rather than intrafraction basis.

The complexity of the observed daily anatomical changes did necessitate increased daily treatment time. Thus, fraction two, which confronted the greatest center of mass change, required a treatment time of 97.6 min, with 67 min spent on CBCT review and re-contouring. Of note, fractions two and five were staffed by a covering physician that day, while all other fractions were covered by the prescribing physician. Challenges encountered when a covering physician was assigned to adaptation may have included varying quality of daily images compared to simulation, lack of familiarity with a particular organ site when staffed by disease site-specific physicians, and ultimately, lack of familiarity with the particular case. We continue to work on strategies to optimize the adaptive treatment workflow in our department, and multiple workflow-based research projects are ongoing to better investigate logistical challenges. Contouring accounted for the greatest increase in time, while other factors such as plan generation, QA, and treatment delivery times were minimally changed.

While we were able to identify the mobility of this target prior to planning due to a comparison of the initial CT and MRI simulation scans, it is possible that other cases with mobile targets could remain unidentified until the first treatment fraction. Furthermore, varying adaptive modalities may allow for different detection of target changes, particularly intra vs. interfraction motion. Other reports utilizing MR-based adaptive planning have presented strategies to manage both inter and intrafraction motions that occur potentially from physiologic processes such as breathing, or peristalsis of the GI tract [18]. It is important to note that the CBCT-based approach in this case allowed primarily for adaptation of interfraction changes. This report adds to the current body of literature demonstrating the potential of adaptive-based treatments to dose-escalate SBRT for oligometastatic disease in the pelvis [19-21]. The use of CBCT adaptive planning, in this case, allowed us to proceed with SBRT for a target that otherwise would not have been amenable to ablative therapy with traditional planning. Ultimately, the ability to re-contour and re-plan based on daily CBCT imaging allowed us to account for significant daily changes in anatomy and to meet the OAR constraints. The patient tolerated treatment without any adverse events.

In sum, there are several points learned from this case that may be useful to consider in future cases. Firstly, it is important to differentiate between intrafraction and interfraction motions. Had we only relied on a 4D-CT sim, negligible intramotion of the target would have been appreciated during treatment planning. However, the significant interfraction motion of the target would only have been noticed at the treatment set-up. In situations where separate CT and MRI simulations are not available, having adequate and well-spaced fiducial markers may be important. This allows for the treatment team to identify potential rotational changes in the target on treatment days. If there is significant rotational change, simple couch shifts may not be sufficient to provide adequate coverage while sparing OARs. From a workflow standpoint, we found that adaptive fractions treated by a covering physician led to significantly longer re-contouring times. Ideally, the prescribing physician is present for all fractions, though this may not be realistic in all departments. To address this, we have established a workbook where treating physicians will document key contouring features on each fraction, which may allow for increased contouring efficiency at the next fraction. The impact of this process implementation is currently being studied in our department.

## Conclusions

We reported a novel application of CBCT-guided OART that allowed for the administration of ablative treatment to an unexpectedly mobile target unamenable to conventional SBRT. The report highlights the potential for unfavorable target motion even in areas typically more stable, such as the pelvis. CBCT-guided OART currently requires high treatment time, a need which might be reduced by optimization of daily contouring. The phase 1 clinical trial NCT05880667 is ongoing and may provide further evidence that CBCT-guided OART can meet the technical challenges posed by radiotherapy for oligometastatic abdominal and pelvic disease.
